# Prognostic impact of angiotensin-converting enzyme inhibitors and angiotensin receptors blockers in esophageal or gastric cancer patients with hypertension - a real-world study

**DOI:** 10.1186/s12885-022-09513-4

**Published:** 2022-04-20

**Authors:** Po-Chih Li, Ru-Yu Huang, Yu-Chien Yang, Kun-Pin Hsieh, Yi-Hsin Yang

**Affiliations:** 1 School of Pharmacy, College of Pharmacy, Kaohsiung Medical University, 100, Shih-Chuan 1st Road, Kaohsiung, Taiwan; 2 Department of Pharmacy, Kaohsiung Chang Gung Memorial Hospital, Kaohsiung, Taiwan; 3 National Institute of Cancer Research, National Health Research Institutes, No.367, Sheng-Li Rd., North District, Tainan, 70456 Taiwan; 4 Department of Pharmacy, Chi Mei Medical Center, Tainan, Taiwan; 5 Department of Pharmacy, Kaohsiung Medical University Hospital, Kaohsiung, Taiwan

**Keywords:** Esophageal cancer, Gastric cancer, Angiotensin-converting enzyme inhibitors (ACEIs), Angiotensin receptor blockers (ARBs), Epidemiology, Survival analysis

## Abstract

**Background:**

Angiotensin-converting enzyme inhibitors (ACEIs) and angiotensin receptor blockers (ARBs) are used in treating cardiovascular diseases. Previous studies indicated that ACEIs/ARBs may benefit cancer patients by inhibiting tumor angiogenesis and proliferation. The effect of ACEIs/ARBs on cancer survival in esophageal and gastric cancer is still unclear. This study is to investigate the association between ACEIs/ARBs usage and esophageal and gastric cancer prognosis.

**Methods:**

This retrospective cohort study identified esophageal and gastric cancer patients during 2008–2016 from the Taiwan Cancer Registry, and obtained medication usage and follow-up information from the National Health Insurance Research Database and Death Registry. Analysis groups were defined as ACEIs/ARBs user or non-user based on the usage of ACEIs/ARBs within the 6 months after cancer diagnosis. The stabilized inverse probability of treatment weighting using propensity scores was applied to balance covariates between study groups. We also used Kaplan-Meier estimates and Cox regression to compare survival outcome and estimate hazard ratios (HRs).

**Results:**

We identified 14,463 and 21,483 newly-diagnosed esophageal and gastric cancer patients during 2008–2016. ACEIs/ARBs users were associated with lower risk of cancer-specific mortality, although only significantly in gastric cancer (gastric: adjusted HR = 0.87, 95% CI = 0.78–0.97; esophageal: adjusted HR =0.88, 95% CI = 0.76–1.02). A better survival outcome was observed among patients who received higher cumulative defined daily dose of ACEIs/ARBs.

**Conclusions:**

We found that using ACEIs/ARBs after cancer diagnosis were associated with lower risk of mortality. Our results add to the knowledge of the benefit of ACEIs/ARBs against mortality in individuals with esophageal/gastric cancer patients with hypertension.

**Supplementary Information:**

The online version contains supplementary material available at 10.1186/s12885-022-09513-4.

## Introduction

Chemoprevention, which is a pharmacological approach to inhibit, delay, or reverse of carcinogenesis before the invasion [1]. Several medications have been widely studied for their potential chemopreventive effect, including statins, metformin, non-steroidal anti-inflammatory drugs (NSAIDs), and angiotensin-converting enzyme inhibitors (ACEIs)/angiotensin receptor blockers (ARBs) [2–8]. In addition to the effect of chemoprevention, the previous study also indicated that using of ACEIs/ARBs may moderately attenuate cancer therapy-related cardiac dysfunction [9]. ACEIs/ARBs are commonly used in treating several cardiovascular diseases, such as hypertension, heart failure, or myocardial infarction (MI) with left ventricular dysfunction [10, 11]. Some studies also indicated that ACEIs/ARBs may have a benefit on cancer prognosis through blocking renin–angiotensin system (RAS) signal pathway and inhibiting tumor angiogenesis and tumor cell proliferation [12, 13]. Previous studies found that most tumor cells have a local RAS mechanism [14]. The information of activated local RAS generating several signals, particularly local angiotensin II (ATII), and augmenting the response of cells in the tumor microenvironment has received substantial attention [15]. Local ATII acting through type 1 angiotensin receptor (AT_1_R) may stimulate tumor cell secretion of numerous cytokines and growth factors into the tumor microenvironment and leading to enhance tumor cell proliferation. ATII also has been demonstrated the association with neovascularization and plays an important role in angiogenesis [16, 17]. Therefore, studies suggested that reduction of the ATII level by RAS inhibitors may also inhibit the angiogenesis and decrease the tumor cell proliferation [18].

Esophageal cancer and gastric cancer are within the top 10 common cancer worldwide [19]. In addition to their high incidence rates, the mortality-incidence ratios are 0.90 and 0.71, respectively (calculated from Table 1 of Sung et al. [19]). Similarly in Taiwan, the mortality-incidence ratios are also relatively high (esophageal: 0.69 & gastric: 0.61) [20] among top 10 cancers. While early detection is still the main strategy to improve survival of cancer patients, the development of possible chemoprevention target may be necessary to improve cancer prognosis.

**Table 1:** Multivariable analysis of cancer-specific mortality in esophageal and gastric cancer

	Esophageal cancer				Gastric cancer			
	Adjusted HR with SIPTW^a,b^	95% CI		*p*-value	Adjusted HR with SIPTW^a,b^	95% CI		*p*-value
**At the post-diagnosis period**								
ACEIs/ARBs non-user	Ref				Ref			
ACEIs/ARBs users	0.88	0.76	1.02	0.097	0.87	0.78	0.97	0.016
**Gender**								
Female	Ref				Ref			
Male	1.04	0.82	1.32	0.756	1.00	0.90	1.11	0.972
**Age group**								
20–49	Ref							
50–64	0.82	0.62	1.09	0.171	1.02	0.74	1.42	0.897
65–74	0.92	0.69	1.23	0.580	1.13	0.81	1.56	0.478
≥ 75	1.06	0.77	1.45	0.724	1.38	0.99	1.90	0.054
**Stage of cancer**								
stage_ ≤ 1	Ref				Ref			
stage_2	2.40	1.70	3.39	<.0001	2.71	2.21	3.32	<.0001
stage_3	3.99	2.83	5.62	<.0001	6.27	5.21	7.55	<.0001
stage_4	5.47	3.83	7.83	<.0001	14.06	11.62	17.01	<.0001
**Surgery**								
No	Ref				Ref			
Yes	0.60	0.51	0.72	<.0001	0.40	0.36	0.45	<.0001
**Radiation therapy**								
No	Ref				Ref			
Yes	0.98	0.79	1.22	0.846	1.20	1.01	1.42	0.043
**Chemotherapy**								
No	Ref				Ref			
Yes	0.81	0.64	1.03	0.086	0.75	0.66	0.85	<.0001
**cDDD at the post-diagnosis period**^**c**^								
Non-user	Ref				Ref			
Low-dose group^d^	0.96	0.82	1.12	0.612	1.06	0.93	1.20	0.377
High-dose group^e^	0.65	0.54	0.78	<.0001	0.65	0.57	0.75	<.0001
**Anti-HTN drugs at the post-diagnosis period**^f^								
Non_ CCBs	Ref				Ref			
CCBs users	1.19	1.01	1.40	0.039	0.91	0.82	1.02	0.105
Non_ Beta-blockers	Ref				Ref			
Beta-blockers users	1.06	0.91	1.24	0.457	1.11	0.99	1.25	0.075
Non_ Diuretics	Ref				Ref			
Diuretics users	1.25	1.07	1.46	0.006	1.52	1.37	1.69	<.0001
Non_ Other anti-HTN	Ref				Ref			
Other anti-HTN users	1.00	0.79	1.27	0.984	1.01	0.85	1.19	0.950

^a^*SIPTW* Stabilize inverse probability of treatment weighting

^b^Adjusted variables included age, gender, year of diagnosis, histology, cancer stage, geographic region, comorbidities (myocardial infarction, congestive heart failure, peripheral vascular disease, cerebrovascular disease, mild liver disease, diabetes, moderate or severe renal disease, diabetes without chronic complication), cancer-related treatment (surgery, radiation therapy, chemotherapy, target therapy), anti-hypertensive medication (calcium channel blockers, beta blockers, diuretics, other-classes antihypertension) and co-medication within 6 months before and after cancer diagnosis (metformin, non-steroidal anti-inflammatory drugs, statins, bisphosphonates, antithrombotic agents)

^c^Cumulative defined daily dose (cDDD) was calculated as each patient received DDD of ACEIs/ARBs at the post-diagnosis period (within 6 months after cancer diagnosis)

^d^Low-dose defined as patients received the cDDD of ACEIs/ARBs at the post-diagnosis period was lower than the median cDDD of ACEIs/ARBs. The median cDDD of ACEIs/ARBs at the post-diagnosis period in esophageal and gastric cancer patients were 113.5 and 122, respectively

^e^High-dose defined as patients received the cDDD of ACEIs/ARBs at the post-diagnosis period was at least the median cDDD of ACEIs/ARBs

^f^*HTN* hypertension, *CCB* calcium channel blocker

Lever et al. conducted a retrospective cohort study which was the first report that long-term use of ACEIs may protect against cancer [21]. A meta-analysis of observational studies also found a decreased risk of cancer associated with the use of ACEIs/ARBs [22]. Recently, a systematic review and meta-analyses found that there was no evidence of an association between ARBs and risk of cancer [23]. However, conflicting results between randomized controlled trials and observational studies were also observed in that meta-analysis. It might be due to different study designs and study qualities. Several studies had also investigated the association between ACEIs/ARBs and cancer prognosis [24–26]. Cardwell et al. conducted a population-based study and found that there was no evidence of increases in cancer-specific mortality in patients who received ACEIs/ARBs after diagnosis of breast cancer, colorectal, or prostate cancer [24]. Recently, two meta-analyses have assessed the potential benefit of ACEIs/ARBs on cancer recurrence and survival and found that using ACEIs/ARBs has a significant reduction of risk of cancer recurrence and mortality [25, 26]. Despite some studies have investigated the association between ACEIs/ARBs and cancer, a limited number of data have focused on esophageal and gastric cancer, particularly esophageal squamous cell carcinoma. To date, the prognosis of esophageal and gastric cancer is still unsatisfactory and the results of antitumor effect of ACEIs/ARBs on esophageal and gastric cancer are controversial. In the present study, we conducted a large scale real-world study to investigate the potential benefit of ACEIs/ARBs on cancer survival in patients with esophageal and/or gastric cancer.

## Methods

### Study design and data sources

We conducted a retrospective cohort study by using population-based databases provided by the Health and Welfare Data Science Center (HWDC), Ministry of Health and Welfare (MOWH), Taiwan [27]. These databases included the Taiwan Cancer Registry (TCR), National Health Insurance Research Database (NHIRD) and Taiwan Death Registry (TDR).

The TCR databases consist of the annual report (AR), long-form (LF) and short-form (SF) data files. The AR data file contains annual cancer incidence cases which are parallel to the Cancer Registry Annual Report announced yearly by Taiwan government [28]. The cancer staging, first course of treatment and follow-up information of selected cancers are collected into the TCR-LF and TCR-SF files. Initially the information of newly diagnosed cancer patients in hospital was reported in TCR-Short Form (TCR-SF). In order to collect more information, TCR-Long Form (TCR-LF) was established and included more detailed information in 2002. The completeness of TCR database was 97.7% in 2011 [29].

The National Health Insurance (NHI) program is a compulsory single-payer health care system which has been implemented since 1995 in Taiwan. At the end of 2009, approximately 23,000,000 people, representing more than 99% of Taiwan population, were enrolled in the NHI program [30]. Recently, according to the National Health Insurance Statistics in Taiwan, more than 90% of hospitals and clinics had a contract with the National Health Insurance Administration. The NHIRD has detailed information of medical services for patients, including registry for beneficiaries, ambulatory care claims, inpatient claims and prescriptions dispensed at pharmacies. We constructed our study cohort by linking the encrypted personal identification (ID) to extract patients’ information, including diagnosis dates, diagnostic code, comorbidities, cancer-related treatment (e.g. surgery, chemotherapy, radiation therapy and chemoradiotherapy), medication, the date and duration of patients receiving therapy and geographic region. Finally, we linked these encrypted ID number to the TDR for survival status as well as date and cause of death.

For privacy protection, only pre-approved researchers are permitted to access these patient-level databases in HWDC. Data management and analyses are all performed within a designated area. To prevent any possibility of recognizing personal identity, HWDC reviews all analysis results, and only statistics computed from cell size of 3 or more are allowed to distribute. The study was conducted according to the guidelines of the Declaration of Helsinki, and approved by the Institutional Review Board of Kaohsiung Medical University Hospital (KMUHIRB-EXEMPT (I)-20,200,056).

### Study cohorts

Two cohorts of patients with newly-diagnosis esophageal (ICD-O-3: C15) or gastric cancer (ICD-O-3: C16) were first identified by using the third edition of International Classification of Diseases for Oncology (ICD-O-3) from the TCR-LF data between January 1, 2008 and December 31, 2016. We only included patients who received their whole cancer treatment within the same hospital (TCR-LF: class = 1 or 2). Patients with aged less than 20 years old or no beneficiary records were excluded. We focused on patients with either adenocarcinoma or squamous cell carcinoma, therefore esophageal cancer patients who were not in the histology of adenocarcinoma (tumor histological codes: 8045, 8140, 8145, 8210, 8211, 8255, 8260, 8263, 8380, 8401, 8480, 8490, 8574) and squamous cell carcinoma (8051, 8052, 8070, 8071, 8072, 8073, 8074, 8075, 8076, 8078, 8083, 8084, 8123), and gastric cancer patients who were not in the histology of adenocarcinoma (8045, 8140, 8141, 8142, 8143, 8144, 8145, 8210, 8211, 8255, 8260, 8261, 8263, 8323, 8480, 8481, 8490, 8500, 8510, 8550, 8551, 8576) and squamous cell carcinoma (8013, 8070, 8071, 8072, 8074, 8076, 8082) were excluded. Patients without staging information or having different diagnosis stages within 28 days were excluded. We excluded patients who had other prior cancer by linking to TCR-AR data, and patients who had 2 cancer registry records at more than 28 days apart in TCR-LF with the same cancer site. This exclusion allowed us to have relatively clear records of primary cancer treatments. Different cardiovascular diseases may have varying degrees of impact on prognosis. To ensure the same indication for ACEIs/ARBs, hypertension was selected as our target indication. Since the laboratory data could not be acquired from our claim database, the diagnostic criteria for hypertension were based on diagnosis code along with medication. To prevent selection bias, we included patients with at least one diagnosis of hypertension (ICD-9-CM: 401; ICD-10-CM: I10) and at least one prescription of the antihypertensive medication within the 6 months before esophageal or gastric cancer diagnosis. To identify the date of death and patients’ status from 2008 to 2017, we linked our cohort to the Multiple Cause of Death Data in the TDR database. The data sources for analysis variables are listed in Supplement Table S1.

### Drug categories and analysis groups

It takes some time for ACEIs/ARBs to have an effect on cancer recurrence and survival [31, 32], and some studies thought a lag-time of 6 months may be appropriate and can prevent reverse causation [33]. We therefore restricted patients who had used ACEIs/ARBs within the 6 months before esophageal or gastric cancer diagnosis to ensure that these patients had similar indications. Based on the use of ACEIs/ARBs within the 6 months after esophageal or gastric cancer diagnosis, patients would be classified into ACEIs/ARBs users and non-users.

There were ten ACEIs reimbursed by the Taiwan NHI program including captopril, enalapril, lisinopril, perindopril, ramipril, quinapril, benazepril, cilazapril, fosinopril, and imidapril. For ARBs, there were eight ARBs had been reimbursed including losartan, eprosartan, valsartan, irbesartan, candesartan, telmisartan, olmesartan, and azilsartan by the Taiwan NHI program. Other antihypertensive medication included beta-blockers (BBs), calcium channel blockers (CCBs), diuretics, and vasodilators. The Anatomical Therapeutic Chemical (ATC) codes for the anti-hypertensive medication were listed in Supplement Table S2.

We used the defined daily dose (DDD) system to test the dose-response relationship between ACEIs/ARBs and cancer prognosis. The DDD system which is described by the World Health Organization is the assumed average maintenance dose per day for a drug used for its main indication in adults [34]. After calculating the cumulative dosage of each ACEIs/ARBs, we then divided this amount by DDD to obtain the cumulative amount of DDD (cDDD) of each ACEIs/ARBs by each patient during a period of 6 months after the diagnosis of esophageal or gastric cancer. Based on the median of cDDD of ACEIs/ARBs, we classified our study cohorts into the low-dosage or high-dosage group as the cDDD they received at a specific period (within the 6 months after cancer diagnosis) was at least or lower than the median cDDD of ACEIs/ARBs to further test the dose-response relationship.

### Outcome measurements and covariates

The outcome measurements were overall survival (OS) and cancer-specific survival in this study. Each patient would be followed form the index date to the death date or the end of the database (December 31, 2017). The index date was defined as the date patients diagnosed with esophageal or gastric cancer indicated by TCR-LF. Diagram illustrating the time frame of our study is shown in Supplement Fig. S1. The analysis covariates included: age, gender, year of diagnosis, histology, stage of cancer, geographic region, comorbidities, cancer treatments, and antihypertensive and co-medication within the 6 months before and after cancer diagnosis. We used the Charlson’s Comorbidity Index (CCI) to evaluate each patients’ comorbidities at the year before esophageal or gastric cancer diagnosis [35]. Cancer treatments included surgery, radiation therapy, chemotherapy, and target therapy. The procedure codes for NHIRD were listed in Supplement Table S3. Moreover, use of co-medication 6 months prior to or 6 months after diagnosis with esophageal or gastric cancer included metformin, NSAIDs, statins, bisphosphonates, and antithrombotic agents (Supplement Table S3). Comorbidities, treatment modalities, and co-medication were all considered dichotomous variables and all of this information was obtained from the NHIRD database.

### Statistical analysis

The baseline characteristics were presented in mean and standard deviations (SD) for continuous variables, and were compared using t-test. We also used frequency distributions for categorical variables and the comparison was conducted using Chi-square tests. The Kaplan-Meier method and log-rank test were used to estimate and compare the OS rates and caner-specific survival rates. To balance the user and non-user groups at baseline, we used the logistic regression with covariates of age, gender, year of diagnosis, histology, comorbidities, cancer stage, geographic region, anti-hypertensive medication, and co-medication to compute the propensity scores. Then, we further applied the propensity scores through the stabilized inverse probability of treatment weighting (SIPTW) to Cox regressions for estimating the risk of all-cause mortality and cancer-related mortality [36]. To investigate the association between mortality and analysis variables, we used Cox regression to estimate the multivariable adjusted hazard ratios (HRs) and 95% confidence intervals (95% CI), with additional covariates, cancer-related treatment (surgery, radiation therapy, chemotherapy, target therapy), anti-hypertensive medication (CCBs, BBs, diuretics, other-classes antihypertension drugs) and co-medication within 6 months before and after cancer diagnosis (metformin, NSAIDs, statins, bisphosphonates, antithrombotic agents). All of the tests were two-sided, and *p*-value less than 0.05 was considered statistically significant. Data management and statistical analysis were performed using the SAS statistical software version 9.4 (SAS Institute Inc., Cary, NC, USA).

### Subgroup and sensitivity analysis

We conducted subgroup analyses to further investigate whether different treatment modalities would influence the results. Treatment modalities included surgery, radiation therapy, and chemotherapy. We also carried out subgroup analysis by histology (gastric adenocarcinoma and esophageal squamous cell carcinoma) and stage of cancer as these differ in pathogenesis or cancer prognosis. To assess if confounding by indication was driving our results, we conducted further subgroup analysis restricted to patients with similar indications by restricting our analysis to patients with MI, congestive heart failure (CHF), diabetes mellitus (DM), or DM complications diagnosis in the year prior to cancer diagnosis. In addition, we carried out a sensitivity analysis restricted to patients who had a follow-up period longer than 6 months after the cancer diagnosis to resolve potential survival bias.

## Results

**Fig. 1:**
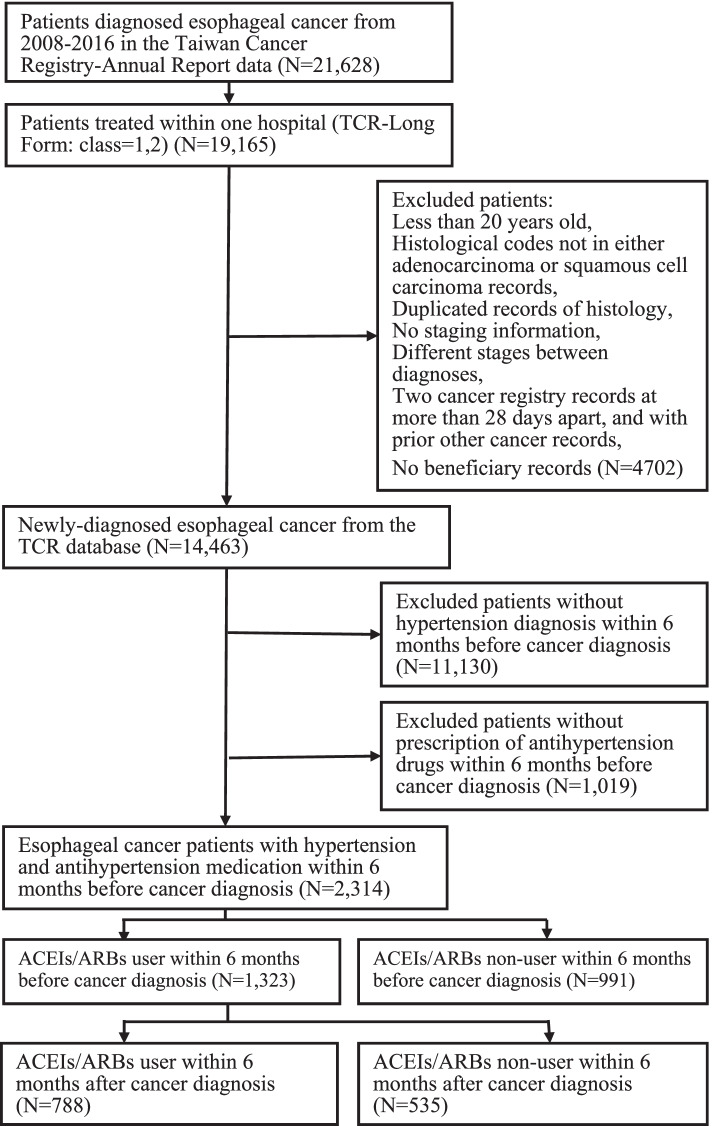
Flowchart of extracting esophageal cancer cohort

**Fig. 2:**
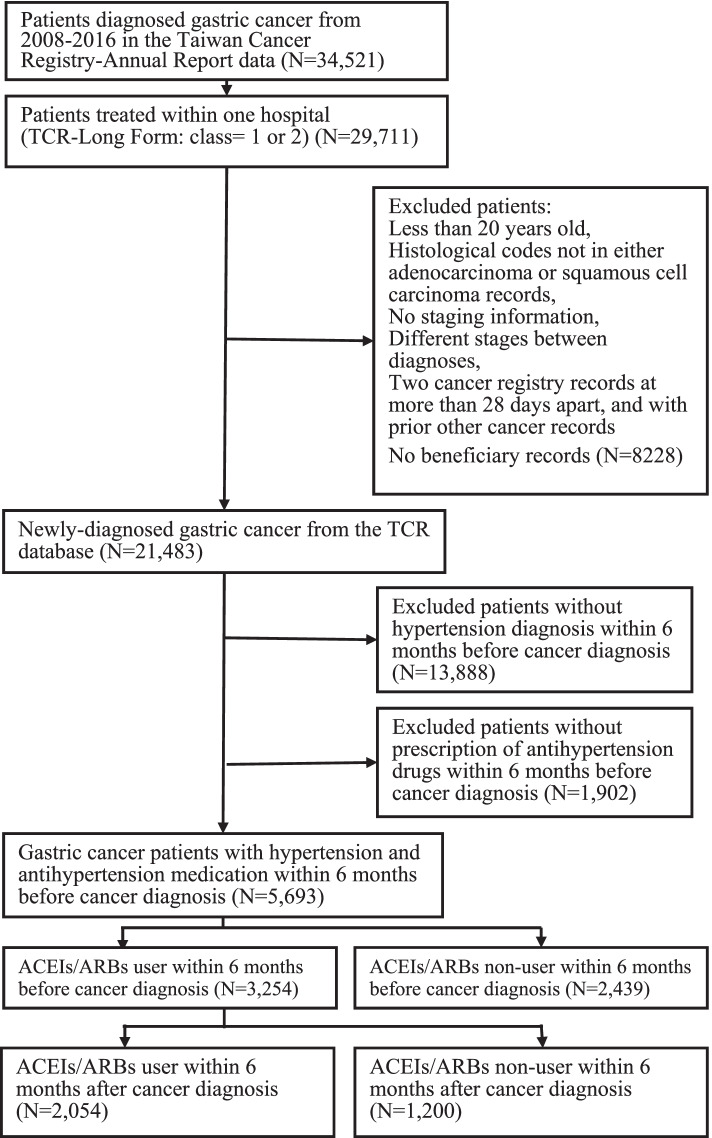
Flowchart of extracting gastric cancer cohort

During the period between January 1, 2008 and December 31, 2016, a total of 19,165 and 29,711 newly diagnosed esophageal and gastric cancer were initially identified from TCR-LF database, respectively. Study flow charts were shown in Figs. 1 and 2.

For esophageal cancer, after preliminary excluded patients who were younger than 20 years old (*N* = 3), patients with neither adenocarcinoma nor squamous cell carcinoma (*N* = 543), patients with duplicated records of histology (*N* = 8), patients who without stage of cancer record (*N* = 312) or with different stage of cancer (*N* = 257), we further excluded patients who had other cancer records prior to the index date (*N* = 3544) and patient with the period between two diagnostic dates more than 28 days (*N* = 32). A total of 14,466 esophageal cancer patients were identified from the TCR-LF database. After identifying patients from TCR-LF, we further excluded patients without NHI database records (*N* = 3), patients with no hypertension diagnosis (*n* = 11,130), and without using the prescription of antihypertensive medications within 6 months before esophageal cancer diagnosis (*N* = 1019). We also exclude patients without using ACEIs/ARBs within the 6 months before esophageal cancer diagnosis (*N* = 991). There were 1323 esophageal cancer patients with ACEIs/ARBs prescriptions including in the analysis. By using similar inclusion and exclusion criteria, a total of 3254 gastric cancer patients with ACEIs/ARBs prescriptions were identified from the TCR-LF and NHIRD databases.

**Fig. 3:**
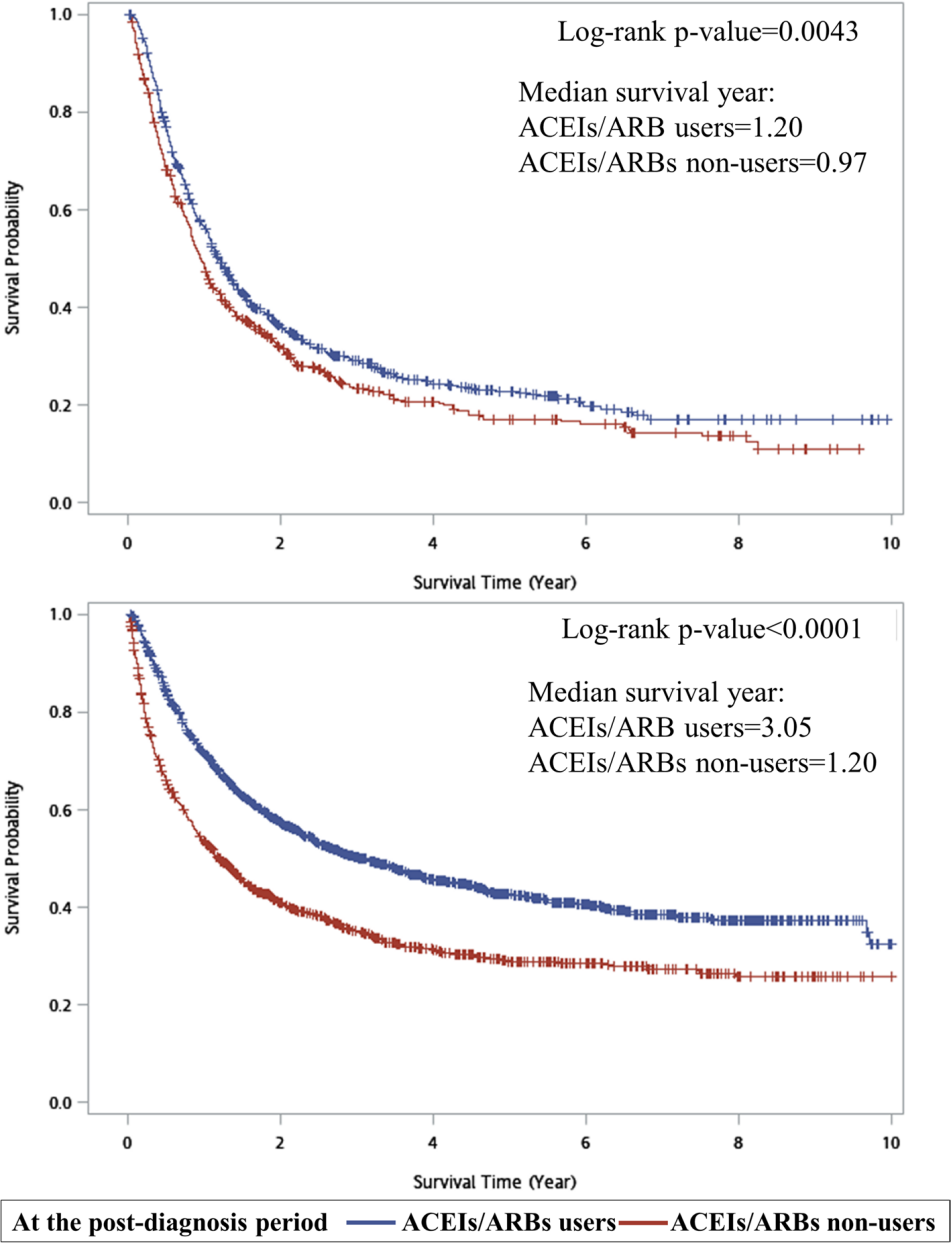
Kaplan-Meier estimates of cancer-specific survival stratified by using of ACEIs/ARBs at the post-diagnosis periods of esophageal cancer (upper) and gastric cancer (lower)

Baseline characteristics for the two groups were shown in Supplement Tables S4 and S5. In esophageal cancer, 788 patients were ACEIs/ARBs users and 535 patients were non-users at the post-diagnosis period (Supplement Table S4). Males accounted for the majority of esophageal cancer, accounting for 90.78%. The mean (±SD) age of esophageal cancer patients was 64.79 (±10.96). About the histology subtypes, over 94% of esophageal cancer patients were squamous cell carcinoma. When considering to stage of cancer, we found that the vast majority in each group were stage 3 or stage 4 esophageal cancer. As shown in Supplement Table S5, 2054 patients were ACEIs/ARBs users and 1200 patients were non-users at the post-diagnosis period in gastric cancer. The mean age of gastric cancer patients was 72.53 (±11.02). We found that gastric cancer patients who used ACEIs/ARBs at the post-diagnosis period had higher CCI scores than non-user (1.24 ± 0.78 and 1.16 ± 0.81; *p* = 0.0026). We also found that adenocarcinoma is the main histology subtype (over 99%) and most gastric cancer patients were stage 3 or stage 4 (56%). Although there were slightly unequal distributions in gender, age stratification, CCI score, stage of cancer between ACEIs/ARBs user and non-user in esophageal cancer or gastric cancer, we found that there were similarly equal distributions after SIPTW weighting. In Fig. 3, we observed that esophageal cancer patients who used ACEIs/ARBs at the post-diagnosis period had better cancer-specific survival than non-users (median survival years: 1.20 vs. 0.97, *p* = 0.0043); similar results were also observed in patients with gastric cancer (median survival years: 3.05 vs. 1.20, *p* < 0.0001).

As shown in Table 1, ACEIs/ARBs was not associated with reduced risk of cancer-specific mortality in patients with esophageal cancer (adjusted HR with SIPTW = 0.88, 95%CI = 0.76–1.02; *p* = 0.097). However, gastric cancer patients who used ACEIs/ARBs at the post-diagnosis period had a 13% decreased risk of cancer-specific mortality than non-users (adjusted HR with SIPTW = 0.87, 95%CI = 0.78–0.97; *p* = 0.016). We found that stage of cancer was the risk factor of cancer-specific mortality. The results of multivariable analysis of all-cause mortality were shown in Supplement Table S6. Table 1 also showed the evidence of a dose-response relationship that patients who received higher cDDD of ACEIs/ARBs at the post-diagnosis period were significantly associated with decreased risk of cancer-specific mortality in both esophageal (adjusted HR with SIPTW = 0.65, 95%CI = 0.54–0.78; *p* < 0.0001) and gastric cancer (adjusted HR with SIPTW = 0.65, 95%CI = 0.57–0.75; *p* < 0.0001). The mortality risk in both cancers was found significant for diuretics users (esophageal: adjusted HR with SIPTW = 1.25, 95%CI = 1.07–1.46; *p* = 0.006); gastric: adjusted HR with SIPTW = 1.52, 95%CI = 1.37–1.69; *p* < 0.0001).

As shown in Table 2, mortality reduction was not significant when restricting to patients who had MI, CHF, DM, or DM with complication records in the year prior to the esophageal cancer diagnosis (adjusted HR with SIPTW = 0.77, 95%CI = 0.58–1.02; *p* = 0.071). However, using ACEIs/ARBs at the post-diagnosis period still had a significantly decreased risk of cancer-specific mortality when we restricted patients with stage 2 (adjusted HR with SIPTW = 0.60, 95%CI = 0.44–0.81; *p* = 0.001), or stage 3 gastric cancer (adjusted HR with SIPTW = 0.79, 95%CI = 0.65–0.96; *p* = 0.020). In addition, there was a significant reduction in cancer-specific mortality among gastric cancer patients who received surgery (adjusted HR with SIPTW = 0.84, 95%CI = 0.73–0.97; *p* = 0.020) or when restricting to patients who received chemotherapy (adjusted HR with SIPTW = 0.83, 95%CI = 0.72–0.96; *p* = 0.012). The results of all-cause mortality were shown in Supplement Table S7.

**Table 2:**
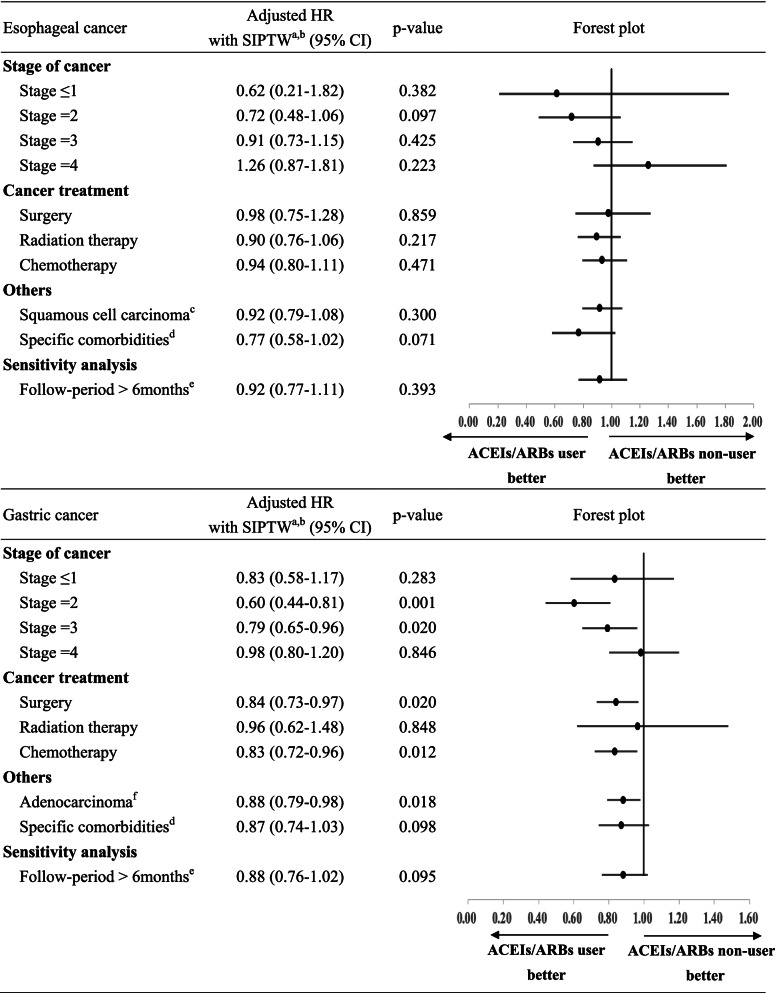
Subgroup and sensitivity analysis of esophageal/gastric cancer mortality

^a^ *SIPTW* Stabilize inverse probability of treatment weighting

^b^Adjusted variables included age, gender, year of diagnosis, histology, cancer stage, geographic region, comorbidities (myocardial infarction, congestive heart failure, peripheral vascular disease, cerebrovascular disease, mild liver disease, diabetes, moderate or severe renal disease, diabetes without chronic complication) cancer-related treatment (surgery, radiation therapy, chemotherapy, target therapy), anti-hypertensive medication (calcium channel blockers, beta blockers, diuretics, other-classes antihypertension) and co-medication within 6 months before and after cancer diagnosis (metformin, non-steroidal anti-inflammatory drugs, statins, bisphosphonates, antithrombotic agents)

^c^ Restricted to patients with esophageal squamous cell carcinoma

^d^ Restricted to patients with myocardial infarction, congestive heart disease, diabetes mellitus or diabetes mellitus with complication in the year prior to the esophageal cancer diagnosis

^e^ Restricted to patients who live longer than 6 months after gastric cancer diagnosis

^f^ Restricted to patients with gastric adenocarcinoma

## Discussion

This large population-based retrospective cohort study found that esophageal and gastric cancer patients who used ACEIs/ARBs at the post-diagnosis period were associated with reduced cancer-specific mortality, especially in gastric cancer, after SIPTW weighting and adjustment of potential confounding factors. This study also found evidence of a dose-response relationship that esophageal and gastric cancer patients who received higher cDDD of ACEIs/ARBs at the post-diagnosis period had better survival outcomes.

Our findings were consistent with previous studies in gastric cancer [25, 33, 37]. A single-center study demonstrated that ACEIs/ARBs were associated with better OS in advanced gastric cancer patients (HR = 0.55, 95%CI = 0.31–0.97) [37]. Recently, a population-based cohort study conducted by Busby et al. indicated that there was a 17% reduction in cancer-specific mortality among ARBs users [33]. Their study results showed a trend but insignificant of lower mortality risk in gastric cancer (adjusted HR = 0.79, 95%CI = 0.62–1.00) than esophageal cancer (adjusted HR = 0.89, 95%CI = 0.71–1.10). There is evidence that RAS components are overexpressed in a variety of cancer cell types and tissues including gastrointestinal malignancies [38, 39]. Studies have shown that as compared to gastric mucosa of *Helicobacter pylori* (*H. pylori*) negative subjects, RAS components such as AT1R protein expression was 3–4 times higher in *H. pylori* positive subjects [39]. As we know, *H. pylori* is one of the major predictors of gastric carcinogenesis. One animal model reported that in the process of infection time, the level of AT1R obviously increased in the gastric corpus during the chronic phase [40]. This finding implied the influence of AT1R expression in the infiltration and atrophy of gastric mucosal inflammatory cells, as well as the potential for the development of gastric cancer. We speculated that inhibition of ACEIs/ARBs on RAS are beneficial for gastric cancer related to histories of severe gastric mucosal atrophy or *H. pylori* infection. Besides, other studies had reported the association between ATII and stress induces acute gastric mucosal injury, and using ARBs could be of therapeutic benefit for stress-induced gastric injury [41]. In our study, about 31% gastric cancer patients had ulcer disease history. This may explain the significant benefits for gastric cancer rather than for esophageal cancer. Another single center study found that there was no significant difference in OS (HR = 0.66, 95%CI = 0.35–1.25) or disease-free survival (HR = 0.75, 95%CI = 0.42–1.34) between the ACEIs/ARBs users and non-users among esophagectomy patients with esophageal cancer [42]. Nevertheless, our results of no significant benefit in esophageal cancer were conflicted with the finding by Chen et al. [43]. Their results showed that use of ACEIs/ARBs (OR = 0.28, 95%C = 0.11–0.70) was an independent prognostic factor of OS in patients with esophageal squamous cell carcinoma. This difference may come from that the included only patients receiving esophagectomy, and hence most of their patients were in stages 1 2. Since majority of our esophageal cancer patients were in late-stage, they might have a shorter follow-up duration. Therefore, the specific mechanism of ACEIs/ARBs in esophageal cancer remains unclear, and the results of human studies are still controversial. More studies are still required to clarify the relationship.

In dose-response analysis, we observed a dose-response relationship between ACEIs/ARBs and mortality in both esophageal and gastric cancer. Busby et al. also found evidence of a dose-response relationship with the lowest HRs observed among patients receiving at least 730 DDDs of ARBs (adjusted HR = 0.42, 95%CI = 0.25–0.72) [33]. Although the high-dose group in our study was defined as patients receiving at least the median DDDs of ACEIs/ARBs or more in the specific period. These results also demonstrated that patients receiving higher dose of ACEIs/ARBs after the cancer diagnosis might have better survival outcome.

Our study also revealed that patients with early-stage of cancer, surgery, and chemotherapy had significantly better survival outcomes. The results were similar to previous studies. Recently, a study conducted by Cheng et al. indicated that the stage of cancer, age, sex, tumor location, tumor length, and treatment were independent prognosis factors in patients with esophageal cancer [44]. They also indicated that there were better outcomes for those patients who could receive surgery.

As the cancer stage is lower in users than non-users, SIPTW weighting was used to control the imbalance between groups. ACEIs/ARBs users are associated with better survival, particularly in gastric cancer, and the stage was an independent prognosis factor in the main analysis. Furthermore, the benefit in OS of receiving ACEIs/ARBs was observed when restricting to early-stage gastric cancer in our subgroup analysis. Previous studies indicated that angiogenesis is a critical step in the progression of human malignancies [45]. Inhibition of tumor angiogenesis is also the critical importance of the angiogenic switch during early tumor development because it is associated with tumors to grow and continue proliferation [46]. It may be the reason that the benefit of ACEIs/ARBs and cancer prognosis only in patients with early-stage cancer. However, due to limited evidence, we suggest that ACEIs/ARBs may be auxiliary but not replace the major treatment for cancer patients. Evidence revealed that RAS inhibitors combined with chemotherapy or chemo-radiotherapy have beneficial effects in cancers, such as colorectal cancer, gastric cancer, glioblastoma, or lung cancer [47]. Beside anti-angiogensis or inhibition of tumor microenvironment, RAS inhibitors may improve drug and oxygen delivery and potentiates chemotherapy [47, 48]. On the other hand, studies have shown that RAS inhibitor might relief radiation-induced injury. Radiation-induced injury arises from overexpression of ATII, and next to up-regulated the pro-fibrogenic and pro-inflammatory pathways [47]. There were potential benefits in different treatment modalities in conjunction with ACEIs/ARBs in our subgroup analyses, although there were only significant in surgery or chemotherapy in gastric cancer. Different ACEIs/ARBs may have various effect on cancer type, and synergize with treatment modalities to produce different effects. There is still needs to conduct further large-scale study to investigate the association between ACEIs/ARBs and treatment modalities. In the sensitivity analysis, patients who live longer than 6 months after esophageal or gastric cancer diagnosis still had a trend that using ACEIs/ARBs after cancer diagnosis had a better survival benefit than non-users, although the difference was not statistically significant.

Our results showed that diuretics use was associated with increased cancer-specific mortality in both esophageal and gastric cancer. The association between diuretics and cancer prognosis is still controversial [49]. Cui et al. found that diuretics was associated with better stomach-specific survival after considering lag period in analysis [50]. No statistically significant association was reported from studies of thiazides and mortality of digestive cancers [49, 51]. Liu et al. discovered higher esophageal or gastric cancer mortality in furosemide users [52]. As compared to thiazide diuretics, loop diuretics are more frequently prescribed to heart failure, and severe liver or kidney diseases related fluid retention. Therefore, this may imply that loop diuretics users tend to have worse health condition. Although we found that diuretics users have higher mortality risk, different types of diuretics may need to be separately investigated, and future study should be conducted to clarify their mortality risks.

There are several histopathological subtypes of esophageal and gastric cancer. The adenocarcinoma of esophagus was predominated in developed Western countries [53, 54], while in Taiwan, the majority was squamous cell carcinoma. As for gastric cancer, adenocarcinoma was the main histopathological subtype. The benefits of ACEIs/ARBs on survival have shown in gastric adenocarcinoma but not in esophageal squamous cell carcinoma in our study. Busby et al. demonstrated that a trend of lower risk on adenocarcinoma than squamous cell carcinoma among gastro-oesophageal cancer, although it was not statistically significant. It should be noted that the histology types were not further classified by cancer type in that study [33].

### Strengths and limitations

To the best of our knowledge, this retrospective cohort study is the first study to investigate ACEIs/ARBs use and survival from esophageal and gastric cancer prognosis by using the population-based database in Taiwan. The TCR database is one of the high-quality cancer registries in the world and TCR-LF registration which involved 80 hospitals counting for over 90% of total cancer cases in Taiwan [29]. The NHIRD database is also one of the largest nationwide population which covered over 99% of residents in Taiwan [30]. By using these databases, we can obtain sufficient information about our study population and with good internal generalizability. Furthermore, we also tested the dose-response relationship and found that patients receiving more cDDD of ACEIs/ARBs after the cancer diagnosis had better survival outcomes. Additionally, some patients had shorter expected survival time that may cause potential selection bias in our study. However, the finding from our sensitivity analysis which restricts those patients who have to live more than 6 months was still observed similar results.

Although there are many strengths, our study still has several potential weaknesses. Although we have adjusted several covariates in multivariable analysis, some factors which may affect the cancer prognosis such as performance status are not available from these databases. We cannot obtain the information for out-of-pocket medications or treatments from our claim database. Because the NHI system covers most of the medical expenses, and ACEIs/ARBs are prescription drugs, we think that patients would prefer to use health insurance rather than self-payment. Therefore, the comprehensive medication records of our study population could be acquired in the claim database. In our results, we also observed patients who belong to ACEIs/ARBs users at the post-diagnosis period were more frequently diagnosed with early-stage than non-users in both esophageal and gastric cancer. It could be a potential healthy survivor bias in our study. However, after the SIPTW weighting, the difference was not statistically significant. We thought it has a limited impact on our research results.

## Conclusions

By using the population-based real-world databases, we found that using ACEIs/ARBs after the cancer diagnosis is significantly associated with better survival outcomes. Our results showed that there was a significant reduction in cancer-specific mortality among gastric cancer patients using ACEIs/ARBs during the post-diagnosis period (HR = 0.87, 95%CI = 0.78–0.97). There was a slightly stronger association among gastric adenocarcinoma than esophageal squamous cell carcinoma. In addition, we found that esophageal and gastric cancer patients who received higher cDDD of ACEIs/ARBs at the post-diagnosis period had significantly decreased risk of mortality. Our results add to the knowledge of the benefit of ACEIs/ARBs on esophageal/gastric cancer prognosis.

## Supplementary Information

**Additional file 1.**

## Data Availability

The data that support the findings of this study are available from Health and Welfare Data Science Center, Ministry of Health and Welfare but restrictions apply to the availability of these data, which were used under license for the current study, and so are not publicly available. Data are however available from the authors upon reasonable request and with permission of Health and Welfare Data Science Center, Ministry of Health and Welfare, Taiwan.
