# Supplementary material for: Prognostic impact of angiotensin-converting enzyme inhibitors and angiotensin receptors blockers in esophageal or gastric cancer patients with hypertension - a real-world study
Source: BMC Cancer. 2022 Apr 20;22:430. doi: 10.1186/s12885-022-09513-4 (PMC9022235; doi:10.1186/s12885-022-09513-4)
Supplement: Supplementary file 1 — Additional file 1. [file 12885_2022_9513_MOESM1_ESM.docx]

Prognostic Impact of Angiotensin-Converting Enzyme Inhibitors and Angiotensin Receptors Blockers in Esophageal or Gastric cancer Patients with Hypertension - a real-world study

**Supplementary**

**Authors:**

Po-Chih Li^1,2^; Ru-Yu Huang^3^; Yu-Chan Yang^1,4^; Kun-Pin Hsieh^1,5*^; Yi-Hsin Yang^1,3*^

^1^ School of Pharmacy, College of Pharmacy, Kaohsiung Medical University, Kaohsiung, Taiwan

^2^ Department of Pharmacy, Kaohsiung Chang Gung Memorial Hospital, Kaohsiung, Taiwan

^3^ National Institute of Cancer Research, National Health Research Institutes, Tainan, Taiwan

^4^ Department of Pharmacy, E-Da Hospital, Kaohsiung, Taiwan

^5^ Department of Pharmacy, Kaohsiung Medical University Hospital, Kaohsiung, Taiwan

^*^ Corresponding authors (equally contribution):

Yi-Hsin Yang, No.367, Sheng-Li Rd., North District, Tainan, 70456 Taiwan; Tel: +886-6-700-0123 ext. 65111; Fax: +886-6-208-3427; Email: [yhyang@nhri.edu.tw](mailto:yhyang@nhri.edu.tw)

Kun-Pin Hsieh, School of Pharmacy, College of Pharmacy, Kaohsiung Medical University, 100, Shih-Chuan 1st Road, Kaohsiung, Taiwan; E-mail: [kphsieh@kmu.edu.tw](mailto:kphsieh@kmu.edu.tw)

**Supplementary Materials:**

Table S1: Data sources of variables used in this study

Table S2: ATC code for study drug included in this study

Table S3: NHI Procedure codes for esophageal / gastric cancer

Table S4: Baseline characteristics before/after SIPTW weighting in esophageal cancer

Table S5: Baseline characteristics before/after SIPTW weighting in gastric cancer

Table S6: Multivariable analysis of all-cause mortality in esophageal and gastric cancer

Table S7: Subgroup and sensitivity analysis of all-cause mortality in esophageal/gastric cancer

Figure S1: Diagram of study time frame and demonstration of patients’ follow-up

Table S1: Data sources of variables used in this study

| Variable | Database | Dataset | Variable |
| --- | --- | --- | --- |
| Age | TCR | Long Form | DIAGAGE |
| Cancer type | TCR | Long Form | CASITE |
| Class | TCR | Long Form | Class |
| Diagnosis date | TCR | Long Form | DIAG_DT |
| Gender | TCR | Long Form | ID_S |
| Histology | TCR | Long Form | HIST |
| Stage | TCR | Long Form | CSTAGE, PSTAGE |
| Smoking status | TCR | Long Form | SMOKING |
| Drink status | TCR | Long Form | DRINK |
| *Helicobacter pylori* status | TCR | Long Form | SSF3 |
| Surgery | TCR | Long Form | OPTYPE, OPTYPE_O |
| Radiation therapy | TCR | Long Form | RTSUMM |
| Chemotherapy | TCR | Long Form | CH, CH_O |
| Target therapy | TCR | Long Form | TARGET_H, TARGET_O |
| Admission date | NHI | Ambulatory Care Expenditures by Visits Inpatient Expenditures by Admissions | FUNC_DATE  IN_DATE |
| Medication | NHI | Details of Ambulatory Care Orders  Details of Inpatient Order  Details of Prescriptions Dispensed at Contracted Pharmacies | DRUG_NO  ORDER_CODE  DRUG_NO |

✥ TCR=Taiwan Cancer Registry Database; NHI=National Health Insurance database; DR=Death Registry

Table S1: Data sources of variables used in this study (continued)

| Variable | Database | Dataset | Variable |
| --- | --- | --- | --- |
| Quantity of medication | NHI | Details of Ambulatory Care Orders  Details of Inpatient Orders  Details of Ambulatory Care Orders Details of Inpatient Order | TOTAL_Q  ORDER_Q  TOTAL_Q |
| Days of administration | NHI | Details of Ambulatory Care Orders  Details of Ambulatory Care Orders Details of Inpatient Order | DRUG_DAY  DRUG_DAY |
| Case classification | NHI | Details of Ambulatory Care Orders  Details of Ambulatory Care Orders Details of Inpatient Order | CASE_TYPE  CASE_TYPE |
| Comorbidities | NHI | Ambulatory Care Expenditures by Visits Inpatient Expenditures by Admissions | ICD9CM_1, ICD9CM_2, ICD9CM_3  ICD9CM_1, ICD9CM_2, ICD9CM_3, ICD9CM_4, ICD9CM_5 |
| Geographic region | NHI | Registry for Beneficiaries | ID1_DIVISION |
| Date of death | TDR | Multiple Cause of Death Data | D_DATE |

✥ TCR=Taiwan Cancer Registry Database; NHI=National Health Insurance database; TDR=Taiwan Death Registry

Table S2: ATC code for study drug included in this study

| **Drug categories** | **Drug name** | **ATC code** |
| --- | --- | --- |
| Angiotensin- converting enzyme inhibitors (ACEIs) | Benazepril | C09AA07, C09BB |
|  | Captopril | C09AA01, C09BA01 |
|  | Cilazapril | C09AA08 |
|  | Enalapril | C09AA02, C09BA02, C09BB |
|  | Fosinopril | C09AA09 |
|  | Imidapril | C09AA16 |
|  | Lisinopril | C09AA03 |
|  | Perindopril | C09AA04, C09BA04, C09BB04 |
|  | Quinapril | C09AA06 |
|  | Ramipril | C09AA05, C09BB05 |
| Angiotensin receptor blockers (ARBs) | Azilsartan | C09CA09, C09DA09 |
|  | Candesartan | C09CA06, C09DA06, C09DB07 |
|  | Eprosartan | C09CA02 |
|  | Irbesartan | C09CA04, C09DA04 |
|  | Losartan | C09CA01, C09DA01 |
|  | Olmesartan | C09CA08, C09DA08, C09DB02, C09DX03 |
|  | Telmisartan | C09CA07, C09DA07, C09DB04 |
|  | Valsartan | C09CA03, C09DA03, C09DB01, C09DX01, C09DX04 |
| Beta blockers (BBs) | Acebutolol | C07AB04 |
|  | Alprenolol | C07AA01 |
|  | Atenolol | C07AB03, C07BB03 |
|  | Betaxolol | C07AB05, S01ED02 |

Table S2. ATC code for study drug included in this study (continued)

| **Drug categories** | **Drug name** | **ATC code** |
| --- | --- | --- |
| Beta blockers (BBs) | Bisoprolol | C07AB07 |
|  | Bupranolol | C07AA19 |
|  | Carteolol | C07AA15, S01ED05 |
|  | Esmolol | C07AB09 |
|  | Metoprolol | C07AB02, C07BB02 |
|  | Nadolol | C07AA12 |
|  | Nebivolol | C07AB12 |
|  | Oxprenolol | C07AA02 |
|  | Pindolol | C07AA03, C07CA03 |
|  | Propranolol | C07AA05 |
|  | Sotalol | C07AA07 |
|  | Timolol | C07AA06, S01ED01 |
| Calcium channel blockers (CCBs) | Amlodipine | C08CA01, C09BB04, C09DB01, C09DB02, C09DB04, C09DB07, C09DX01, C09DX03, C09XA, C09XA52 |
|  | Barnidipine | C08CA12 |
|  | Benidipine | C08CA15 |
|  | Diltiazem | C08DB01 |
|  | Felodipine | C08CA02, C09BB, C09BB05 |
|  | Isradipine | C08CA03 |
|  | Lacidipine | C08CA09 |
|  | Lercanidipine | C08CA13 |
|  | Nicardipine | C08CA04 |
|  | Nifedipine | C08CA05 |

Table S2. ATC code for study drug included in this study (continued)

| **Drug categories** | **Drug name** | **ATC code** |
| --- | --- | --- |
| Calcium channel blockers (CCBs) | Nimodipine | C08CA06 |
|  | Nitrendipine | C08CA08 |
|  | Verapamil | C08DA01 |
| Diuretics | Amiloride | C03DB01, C03EA01, C07DA06 |
|  | Bendroflumethiazide | C03AA01 |
|  | Bumetanide | C03CA02 |
|  | Canrenoate | C03DA |
|  | Clofenamide | C02LA01 |
|  | Clopamide | C02LA51, C07CA03 |
|  | Cyclopenthiazide | C03AA07 |
|  | Eplerenone | C03DA04 |
|  | Furosemide | C02LA01, C03CA01 |
|  | Hydrochlorothiazide | C02LA01, C02LA51, C02LB01, C02LG02, C03AA03, C03EA01, C07BB02, C07DA06, C09BA01, C09BA02, C09DA01, C09DA03, C09DA04, C09DA06, C09DA07, C09DA08, C09DB04, C09DX01, C09DX03, C09XA52 |
|  | Hydroflumethiazide | C03EA |
|  | Indapamide | C03BA11, C09BA04 |
|  | Metolazone | C03BA08 |
|  | Spironolactone | C03DA01, C03EA01, C03EA |
|  | Triamterene | C03DB02, C03EA01 |
|  | Trichlormethiazide | C03AA06 |

Table S2. ATC code for study drug included in this study (continued)

| **Drug categories** | **Drug name** | **ATC code** |
| --- | --- | --- |
| Other classes of antihypertensive medications | Clonidine | C02AC01, N02CX02 |
|  | Doxazosin | C02CA04 |
|  | Guanethidine | C02CC02 |
|  | Hydralazine | C02DB02, C02LA51, C02LG02, C02N |
|  | Methyldopa_levorotatory | C02AB01 |
|  | Methyldopa_racemic | C02AB02 |
|  | Methyldopa | C02LB01 |
|  | Minoxidil | C02DC01 |
|  | Nitroprusside | C02DD01 |
|  | Prazosin | C02CA01 |
|  | Rescinnamine | C02AA01, C02LA50 |
|  | Reserpine | C02AA02, C02LA01, C02LA50, C02LA51, C02N |

Table S3: NHI Procedure codes for esophageal / gastric cancer

| **NHI code** | **Procedure for esophageal cancer** |
| --- | --- |
| 71201B | Esophageal myomectomy |
| 71202B | Excision of esophageal diverticulum |
| 71203C | Endoesophageal intubation |
| 71204B | Esophagofundostomy bypass |
| 71205B | Esophagofundostomy |
| 71206B | Esophagogastrostomy bypass |
| 71207B | Retrograde esophageal dilatation (esophagectasia, retrograde) |
| 71208B | Esophagogastric fistula closure |
| 71209B | Esophagectomy |
| 71210B | Esophagectomy & reconstruction |
| 71211B | Esophagotomy (transcervicle or transthoracic) |
| 71212B | Excision of esophageal cyst & tumor |
| 71213B | Esophageal reconstruction with gastric tube |
| 71220B | Esophageal reconstruction with colon |
| 71221B | Esophageal reconstruction with small intestine |
| 71214B | Repair of esophageal laceration |
| 71215B | Simple excision of esophageal cancer, with lymphadenectomy |
| 71222B | Complicated excision of esophageal cancer, with lymphadenectomy |
| 71216B | Ligation of esophageal varices, transthoracic or transabdominal |
| 71217B | Devascularization procedure: transthoracic |
| 71218B | Devascularization procedure: transabdominal |
| 71219B | Esophagogastric stent for esophagus or cardia portion cancer |
| 71223B | Thoracoscopic Excision of Esophageal Cyst and Tumor |
| 71224B | Thoracoscopic Esophagectomy |
| 71225B | Thoracoscopic or Laparoscopic Esophagomyotomy (Heller myotomy) |

Table S3. NHI Procedure codes for esophageal / gastric cancer (continued)

| **NHI code** | **Procedure for gastric cancer** |
| --- | --- |
| 72001B | Gastrotomy: exploration |
| 72002B | Gastrotomy: removal of foreign body |
| 72042B | Gastrotomy: with suture repair of bleeding ulcer |
| 72003B | Pyloromyotomy, Fredet-Ramstedt |
| 72006B | Local excision, ulcer or tumor |
| 72007B | Gastrectomy, total & angreconstruction |
| 72008B | Gastrostomy & pyloroplasty |
| 72009B | Subtotal gastrectomy or hemigastrectomy with gastro-duodenostomy: without vagotomy |
| 72043B | Subtotal gastrectomy or hemigastrectomy with gastrojejunostomy: without vagotomy |
| 72044B | Subtotal gastrectomy or hemigastrectomy with Roux-en-Y gastrojejunostomy: without vagotomy |
| 72010B | Gastrectomy, subtotal or hemigastrectomy: with vagotomy |
| 72011B | Vagotomy and pyloroplasty |
| 72012B | Pyloroplasty |
| 72013B | Gastro-duodenostomy |
| 72014B | Gastrojejunostomy |
| 72015B | Gastroenterostomy |
| 72016B | Gastrojejunostomy with vagotomy |
| 72017C | Gastrostomy |
| 72018B | Duodenorrhpahy, suture of perforated ulcer |
| 72019B | Gastrorrhaphy, suture or repair wound, injury perforated ulcer of stomach |
| 72020B | Revision of gastroduodenostomy with or without vagotomy |
| 72021B | Re-exploration for postgastrectomy bleeding |
| 72022C | Closure of gastrostomy |
| 72023B | Duodenostomy |
| 72024B | Excision of duodenum tumor |
| 72025B | Excision or inversion of duodenal diverticulum |
| 72026B | Closure of duodenal fistula |
| 72027B | Duodenal obstruction |
| 72028B | Highly selective vagotomy |
| 72029B | Vagotomy |
| 72030B | Proximal gastrectomy & esophagectomy & reconstruction |

Table S3. NHI Procedure codes for esophageal / gastric cancer (continued)

| **NHI code** | **Procedure for gastric cancer** |
| --- | --- |
| 72031B | Gastrectomy, total, with splenectomy or partial pancreatectomy |
| 72032B | Total gastrectomy, with LN dissection, with reconstruction (any type) |
| 72046B | Near total gastrectomy, with LN dissection, with reconstruction |
| 72047B | Radical subtotal gastrectomy, with reconstruction |
| 72033B | Revision of gastrojejunostomy |
| 72034B | Resection of retained antrum, postgastrectomy |
| 72035B | Gastric partition |
| 72036B | Transduodenal sphinteroplasty |
| 72037B | Plication of stomach |
| 72038B | Gastropexy for gastric volvulus |
| 72039B | EPT (endoscopic papillectomy) |
| 72040B | Belsy’s mark iv anti-reflux procedure |
| 72041B | Laparoscopic gastric partition |
| 72045C | Laparoscopic gastrostomy |
| 72048B | Laparoscopic subtotal gastrectomy |
| 72049B | Laparoscopic valgotomy and drainage |
| 72050B | Endoscopic mucosal resection |

Table S4: Baseline characteristics before/after SIPTW^a^ weighting in esophageal cancer

|  |  | | Before SIPTW weighting | | | | | | | | After SIPTW weighting | | | | |
| --- | --- | --- | --- | --- | --- | --- | --- | --- | --- | --- | --- | --- | --- | --- | --- |
|  | Total population | | Non-users^b^ | | Users^c^ | | p-value | | SDiff^d,e^ | | Non-users^b^ | | Users^c^ | p-value | SDiff^d,e^ |
| **N (%)** | 1323 | | 535 | | 788 | |  | |  | | 532 | | 791 |  |  |
| **Gender** |  | |  | |  | |  | |  | |  | |  |  |  |
| Male | 1201 (90.78) | | 497 (92.90) | | 704 (89.34) | | 0.0282 | | 0.1254 | | 481 (90.35%) | | 718 (90.78%) | 0.7910 | 0.0147 |
| Female | 122 (9.22) | | 38 (7.10) | | 84 (10.66) | |  | |  | | 51 (9.65) | | 73 (9.22) |  |  |
| **Age** |  | |  | |  | |  | |  | |  | |  |  |  |
| Mean±SD | 64.79±10.96 | | 63.40±10.90 | | 65.73±10.90 | | 0.0001 | | -0.2137 | | 65.01±11.10 | | 64.88±10.95 | 0.8316 | 0.0118 |
| 20-49 | 88 (6.65) | | 49 (9.16) | | 39 (4.95) | | 0.0003 | | 0.1650 | | 37 (7.03) | | 56 (7.10) | 0.9837 | -0.0027 |
| 50-64 | 612 (46.26) | | 261 (48.79) | | 351 (44.54) | |  | | 0.0853 | | 238 (44.64) | | 359 (45.41) |  | -0.0155 |
| 65-74 | 360 (27.21) | | 142 (26.54) | | 218 (27.66) | |  | | -0.0252 | | 147 (27.66) | | 219 (27.69) |  | -0.0007 |
| ≥75 | 263 (19.88) | | 83 (15.51) | | 180 (22.84) | |  | | -0.1870 | | 110 (20.67) | | 157 (19.81) |  | 0.0214 |
| **Alcohol** |  | |  | |  | |  | |  | |  | |  |  |  |
| Missing | 360 (27.21) | | 153 (28.60) | | 207 (26.27) | | 0.3495 | | 0.0522 | | 143 (26.92) | | 216 (27.26) | 0.9982 | -0.0077 |
| Never | 245 (18.52) | | 87 (16.26) | | 158 (20.05) | |  | | -0.0984 | | 96 (18.01) | | 144 (18.17) |  | -0.0042 |
| Former | 218 (16.48) | | 91 (17.01) | | 127 (16.12) | |  | | 0.0239 | | 92 (17.25) | | 136 (17.22) |  | 0.0008 |
| Current | 500 (37.79) | | 204 (38.13) | | 296 (37.56) | |  | | 0.0118 | | 201 (37.82) | | 295 (27.26) |  | 0.2268 |
| **Smoking** | |  | |  | |  | |  | |  | |  |  |  |  |
| Missing | | 480 (36.28) | | 196 (36.64) | | 284 (36.04) | | 0.0969 | | 0.0125 | | 195 (36.60) | 288 (36.48) | 0.9831 | 0.0025 |
| Never | | 240 (18.14) | | 85 (15.89) | | 155 (19.67) | |  | | -0.0990 | | 95 (17.83) | 142 (18.02) |  | -0.0050 |
| Former | | 89 (6.73) | | 30 (5.61) | | 59 (7.49) | |  | | -0.0760 | | 39 (7.31) | 53 (6.76) |  | 0.0215 |
| Current | | 514 (38.85) | | 224 (41.87) | | 290 (36.80) | |  | | 0.1039 | | 204 (38.26) | 306 (38.74) |  | -0.0099 |
| **Histology** | |  | |  | |  | |  | |  | |  |  |  |  |
| Adenocarcinoma | | 77 (5.82) | | 34 (6.36) | | 43 (5.46) | | 0.4934 | | 0.0382 | | 28 (5.23) | 42 (5.32) | 0.9436 | -0.0040 |
| Squamous cell carcinoma | | 1246 (94.18) | | 501 (93.64) | | 745 (94.54) | |  | |  | | 505 (94.77) | 749 (94.68) |  |  |

Table S4: Baseline characteristics before/after SIPTW^a^ weighting in esophageal cancer (continued)

|  |  | | Before SIPTW weighting | | | | | | | | After SIPTW weighting | | | | | | |
| --- | --- | --- | --- | --- | --- | --- | --- | --- | --- | --- | --- | --- | --- | --- | --- | --- | --- |
|  | Total population | | Non-users^b^ | | Users^c^ | | p-value | | SDiff^d,e^ | | Non-users^b^ | | Users^c^ | | p-value | | SDiff^d,e^ |
| **CCI score** |  | |  | |  | |  | |  | |  | |  | |  | |  |
| Mean±SD | 0.96±0.81 | | 0.93±0.83 | | 0.98±0.80 | | 0.2780 | | -0.0613 | | 0.97±0.82 | | 0.96±0.82 | | 0.8626 | | 0.0122 |
| 0 | 466 (35.22) | | 206 (38.50) | | 260 (32.99) | | 0.0436 | | 0.1152 | | 189 (35.43) | | 279 (35.28) | | 0.9464 | | 0.0031 |
| 1 | 446 (33.71) | | 161 (30.09) | | 285 (36.17) | |  | | -0.1294 | | 173 (32.51) | | 264 (33.33) | |  | | -0.0175 |
| ≥2 | 411 (31.07) | | 168 (31.40) | | 243 (30.84) | |  | | 0.0121 | | 171 (32.06) | | 248 (31.40) | |  | | 0.0142 |
| **Stage of cancer** |  | |  | |  | |  | |  | |  | |  | |  | |  |
| ≤1 | 138 (10.43) | | 57 (10.65) | | 81 (10.28) | | 0.0093 | | 0.0121 | | 51 (9.59) | | 79 (9.98) | | 0.9877 | | -0.0131 |
| 2 | 270 (20.41) | | 85 (15.89) | | 185 (23.48) | |  | | -0.1918 | | 114 (21.41) | | 164 (20.71) | |  | | 0.0172 |
| 3 | 577 (43.61) | | 246 (45.98) | | 331 (42.01) | |  | | 0.0800 | | 235 (44.09) | | 349 (44.20) | |  | | -0.0022 |
| 4 | 338 (25.55) | | 147 (27.48) | | 191 (24.24) | |  | | 0.0740 | | 133 (24.91) | | 199 (25.11) | |  | | -0.0046 |
| Geographic region | |  | |  | |  | |  | |  | |  | |  | |  |  |
| Taipei district | | 494 (37.34) | | 202 (37.76) | | 292 (37.06) | | 0.7600 | | 0.0145 | | 198 (37.22) | | 296 (37.48) | | 0.9994 | -0.0054 |
| North district | | 140 (10.58) | | 56 (10.47) | | 84 (10.66) | |  | | -0.0062 | | 56 (10.56) | | 84 (10.62) | |  | -0.0019 |
| Central district | | 266 (20.11) | | 115 (21.50) | | 151 (19.16) | |  | | 0.0582 | | 113 (21.27) | | 162 (20.50) | |  | 0.0189 |
| South district | | 154 (11.64) | | 57 (10.65) | | 97 (12.31) | |  | | -0.0521 | | 60 (11.27) | | 89 (11.31) | |  | -0.0013 |
| Kaohsiung/Pingtung district | | 217 (16.40) | | 82 (15.33) | | 135 (17.13) | |  | | -0.0488 | | 80 (15.11) | | 124 (15.64) | |  | -0.0147 |
| East district | | 52 (3.93) | | 23 (4.30) | | 29 (3.68) | |  | | 0.0317 | | 24 (4.57) | | 35 (4.44) | |  | 0.0063 |

^a^SIPTW=stabilized inverse probability of treatment weighting

^b^Non-users were defined as the patients without using ACEIs/ARBs at the post-diagnosis period (within the 6 months after esophageal cancer diagnosis).

^c^Users were defined as the patients who used ACEIs/ARBs at the post-diagnosis period (within the 6 months after esophageal cancer diagnosis).

^d^SDiff=Standardized difference

^e^Covariates with the absolute values of SDiff > 0.1 represent meaningful differences between groups.

Table S5: Baseline characteristics before/after SIPTW^a^ weighting in gastric cancer

|  |  | | Before SIPTW weighting | | | | | | | | | After SIPTW weighting | | | | | |
| --- | --- | --- | --- | --- | --- | --- | --- | --- | --- | --- | --- | --- | --- | --- | --- | --- | --- |
|  | Total population | | Non-users^b^ | | Users^c^ | | p-value | | SDiff^d,e^ | | | Non-users^b^ | Users^c^ | | p-value | SDiff^d,e^ | |
| **N (%)** | 3254 | | 1200 | | 2054 | |  | |  | | | 1192 | 2057 | |  |  | |
| **Gender** |  | |  | |  | |  | |  | | |  |  | |  |  | |
| Male | 2107 (64.75) | | 792 (66.00) | | 1315 (64.02) | | 0.2544 | | 0.0415 | | | 763 (64.02) | 1327 (64.52) | | 0.7726 | -0.0104 | |
| Female | 1147 (35.25) | | 408 (34.00) | | 739 (35.98) | |  | |  | | | 429 (35.98) | 730 (35.48) | |  |  | |
| **Age** |  | |  | |  | |  | |  | | |  |  | |  |  | |
| Mean±SD | 72.53 ± 11.02 | | 72.45 ± 11.55 | | 72.58 ± 10.69 | | 0.7348 | | -0.011682 | | | 72.65 ± 11.25 | 72.50 ± 10.87 | | 0.7109 | 0.0136 | |
| 20-49 | 79 (2.43) | | 35 (2.92) | | 44 (2.14) | | 0.1573 | | 0.0497 | | | 29 (2.42) | 50 (2.43) | | 0.9936 | -0.0007 | |
| 50-64 | 706 (21.70) | | 272 (22.67) | | 434 (21.13) | |  | | 0.0372 | | | 253 (21.19) | 443 (21.53) | |  | -0.0083 | |
| 65-74 | 888 (27.29) | | 305 (25.42) | | 583 (28.38) | |  | | -0.0668 | | | 330 (27.71) | 562 (27.31) | |  | 0.0090 | |
| ≥75 | 1581 (48.59) | | 588 (49.00) | | 993 (48.34) | |  | | 0.0132 | | | 580 (48.68) | 1002 (48.73) | |  | -0.0010 | |
| **Alcohol** |  | |  | |  | |  | |  | | |  |  | |  |  | |
| Missing | 997 (30.64) | | 336 (28.00) | | 661 (32.18) | | 0.0129 | | -0.0912 | | | 364 (30.56) | 630 (30.61) | | 0.9995 | -0.0011 | |
| Never | 1691 (51.97) | | 635 (52.92) | | 1056 (51.41) | |  | | 0.0302 | | | 622 (52.19) | 1071 (52.06) | |  | 0.0026 | |
| Former | 199 (6.12) | | 71 (5.92) | | 128 (6.23) | |  | | -0.0130 | | | 71 (6.00) | 125 (6.10) | |  | -0.0042 | |
| Current | 367 (11.28) | | 158 (13.17) | | 209 (10.18) | |  | | 0.0932 | | | 134 (11.26) | 231 (11.23) | |  | 0.0009 | |
| **Smoking** | |  | |  | |  | |  | |  |  | | |  |  | |  |
| Missing | | 1228 (37.74) | | 423 (35.25) | | 805 (39.19) | | 0.0413 | | -0.0816 | 450 (37.73) | | | 776 (37.72) | 0.9942 | | 0.0002 |
| Never | | 1532 (47.08) | | 577 (48.08) | | 955 (46.49) | |  | | 0.0319 | 566 (47.51) | | | 971 (47.20) |  | | 0.0062 |
| Former | | 117 (3.60) | | 41 (3.42) | | 76 (3.70) | |  | | -0.0151 | 41 (3.44) | | | 74 (3.59) |  | | -0.0081 |
| Current | | 377 (11.59) | | 159 (13.25) | | 218 (10.61) | |  | | 0.0815 | 135 (11.33) | | | 236 (11.49) |  | | -0.0050 |
| **Histology** | |  | |  | |  | |  | |  |  | | |  |  | |  |
| Adenocarcinoma | | 3235 (99.42) | | 1195 (99.58) | | 2040 (99.32) | | 0.3386 | | 0.0352 | 1184 (99.32) | | | 2045 (99.40) | 0.7766 | | -0.0100 |
| Squamous cell carcinoma | | 19 (0.58) | | 5 (0.42) | | 14 (0.68) | |  | |  | 8 (0.68) | | | 12 (0.60) |  | |  |

Table S5 Baseline characteristics before/after SIPTW^a^ weighting in gastric cancer (continued)

|  |  | | Before SIPTW weighting | | | | | | | | | After SIPTW weighting | | | | | | |
| --- | --- | --- | --- | --- | --- | --- | --- | --- | --- | --- | --- | --- | --- | --- | --- | --- | --- | --- |
|  | Total population | | Non-users^b^ | | Users^c^ | | | p-value | | SDiff^d,e^ | | Non-users^b^ | | Users^c^ | | p-value | | SDiff^d,e^ |
| **CCI score** |  | |  | |  | | |  | |  | |  | |  | |  | |  |
| Mean±SD | 1.21 ± 0.80 | | 1.16 ± 0.81 | | 1.24 ± 0.78 | | | 0.0026 | | -0.1006 | | 1.21 ± 0.80 | | 1.21 ± 0.80 | | 0.9699 | | 0 |
| 0 | 759 (23.33) | | 318 (26.50) | | 441 (21.47) | | | 0.0042 | | 0.1180 | | 277 (23.22) | | 474 (23.05) | | 0.9935 | | 0.0040 |
| 1 | 1053 (32.36) | | 378 (31.50) | | 675 (32.86) | | |  | | -0.0291 | | 385 (32.34) | | 666 (32.36) | |  | | -0.0004 |
| ≥2 | 1442 (44.31) | | 504 (42.00) | | 938 (45.67) | | |  | | -0.0740 | | 530 (44.45) | | 917 (44.59) | |  | | -0.0028 |
| **Stage of cancer** |  | |  | |  | | |  | |  | |  | |  | |  | |  |
| ≤1 | 892 (27.41) | | 244 (20.33) | | 648 (31.55) | | | <.0001 | | -0.2581 | | 315 (26.43) | | 559 (27.17) | | 0.9650 | | -0.0167 |
| 2 | 540 (16.59) | | 189 (15.75) | | 351 (17.09) | | |  | | -0.0362 | | 196 (16.47) | | 341 (16.57) | |  | | -0.0027 |
| 3 | 841 (25.85) | | 326 (27.17) | | 515 (25.07) | | |  | | 0.0478 | | 310 (26.04) | | 532 (25.86) | |  | | 0.0041 |
| 4 | 981 (30.15) | | 441 (36.75) | | 540 (26.29) | | |  | | 0.2266 | | 370 (31.06) | | 625 (30.41) | |  | | 0.0141 |
| History of peptic ulcer | | 998 (30.67) | | 363 (30.25) | | 635 (30.92) | 0.6913 | | 0.2958 | | 374 (31.36) | | 620 (30.14) | | 0.04637 | | 0.3071 | |
| *H.pylori* infection^f^ | |  | |  | |  |  | |  | |  | |  | |  | |  | |
| Positive | | 505 (15.52) | | 176 (14.67) | | 329 (16.02) | 0.5734 | | -0.0375 | | 170 (14.27) | | 336 (16.34) | | 0.2372 | | -0.0575 | |
| Negative | | 889 (27.32) | | 334 (27.83) | | 555 (27.02) |  | | 0.0182 | | 325 (27.23) | | 567 (27.56) | |  | | -0.0074 | |
| Missing | | 1860 (57.16) | | 690 (57.50) | | 1170 (56.96) |  | | 0.0109 | | 697 (58.50) | | 1154 (56.10) | |  | | 0.0485 | |
| Geographic region | |  | |  | |  |  | |  | |  | |  | |  | |  | |
| Taipei district | | 1341 (41.25) | | 463 (38.58) | | 878 (42.81) | 0.0151 | | -0.0850 | | 489 (41.02) | | 847 (41.24) | | 0.9996 | | -0.0043 | |
| North district | | 414 (12.73) | | 161 (13.42) | | 253 (12.34) |  | | 0.0329 | | 156 (13.09) | | 263 (12.80) | |  | | 0.0086 | |
| Central district | | 450 (13.84) | | 180 (15.00) | | 270 (13.16) |  | | 0.0532 | | 168 (14.09) | | 288 (14.02) | |  | | 0.0026 | |
| South district | | 429 (13.20) | | 163 (13.58) | | 266 (12.97) |  | | 0.0186 | | 152 (12.75) | | 269 (13.10) | |  | | -0.0089 | |
| Kaohsiung/Pingtung district | | 499 (15.35) | | 182 (15.17) | | 317 (15.46) |  | | -0.0072 | | 183 (15.35) | | 313 (15.24) | |  | | 0.0025 | |
| East district | | 118 (3.63) | | 51 (4.25) | | 67 (3.27) |  | | 0.0521 | | 44 (3.69) | | 74 (3.60) | |  | | 0.0059 | |

^a^SIPTW=stabilized inverse probability of treatment weighting

^b^Non-users were defined as the patients without using ACEIs/ARBs at the post-diagnosis period (within the 6 months after gastric cancer diagnosis).

^c^Users were defined as the patients who used ACEIs/ARBs at the post-diagnosis period (within the 6 months after gastric cancer diagnosis).

^d^SDiff=Standardized difference

^e^Covariates with the absolute values of SDiff > 0.1 represent meaningful differences between groups.

^f^*H.pylori* = *Helicobacter pylori*

Table S6: Multivariable analysis of all-cause mortality in esophageal and gastric cancer

|  |  | Esophageal cancer | | | | Gastric cancer | | | |
| --- | --- | --- | --- | --- | --- | --- | --- | --- | --- |
|  |  | Adjusted HR^a^  with SIPTW^b^ | 95%CI | | p-value | Adjusted HR^a^  with SIPTW^b^ | 95%CI | | p-value |
| **At the post-diagnosis period^c^** | | | | | | | | | |
| ACEIs/ARBs non-user | | Ref | | | | Ref | | | |
| ACEIs/ARBs users | | 0.86 | 0.75 | 0.99 | 0.041 | 0.86 | 0.78 | 0.95 | 0.002 |
| **Gender** | |  |  |  |  |  |  |  |  |
| Female | | Ref | | | |  | | | |
| Male | | 1.17 | 0.91 | 1.50 | 0.231 | 1.01 | 0.92 | 1.11 | 0.802 |
| **Age group** | |  |  |  |  |  |  |  |  |
| 20-49 | | Ref | | | |  | | | |
| 50-64 | | 0.84 | 0.64 | 1.10 | 0.200 | 1.08 | 0.78 | 1.48 | 0.659 |
| 65-74 | | 0.97 | 0.73 | 1.28 | 0.806 | 1.20 | 0.87 | 1.64 | 0.268 |
| ≥75 | | 1.13 | 0.83 | 1.54 | 0.426 | 1.67 | 1.22 | 2.29 | 0.001 |
| **Stage of cancer** | |  |  |  |  |  |  |  |  |
| stage_≤1 | | Ref | | | |  | | | |
| stage_2 | | 2.15 | 1.57 | 2.94 | <.0001 | 2.00 | 1.69 | 2.36 | <.0001 |
| stage_3 | | 3.63 | 2.66 | 4.97 | <.0001 | 4.13 | 3.54 | 4.81 | <.0001 |
| stage_4 | | 4.79 | 3.45 | 6.65 | <.0001 | 8.88 | 7.53 | 10.46 | <.0001 |
| **Surgery** | |  |  |  |  |  |  |  |  |
| No | | Ref | | | |  | | | |
| Yes | | 0.57 | 0.48 | 0.67 | <.0001 | 0.42 | 0.38 | 0.47 | <.0001 |
| **Radiation therapy** | |  |  |  |  |  |  |  |  |
| No | | Ref | | | |  | | | |
| Yes | | 0.96 | 0.78 | 1.18 | 0.690 | 1.25 | 1.07 | 1.47 | 0.006 |
| **Chemotherapy** | |  |  |  |  |  |  |  |  |
| No | | Ref | | | |  | | | |
| Yes | | 0.71 | 0.57 | 0.89 | 0.002 | 0.71 | 0.63 | 0.79 | <.0001 |
| **cDDD at the post-diagnosis period^d^** | | |  |  |  |  |  |  |  |
| Non-user | | Ref | | | |  | | | |
| Low-dose group^e^ | | 0.97 | 0.83 | 1.12 | 0.643 | 1.03 | 0.92 | 1.15 | 0.569 |
| High-dose group^f^ | | 0.66 | 0.55 | 0.79 | <.0001 | 0.65 | 0.58 | 0.73 | <.0001 |

^a^Adjusted variables included age, gender, year of diagnosis, histology, cancer stage, geographic region, comorbidities (myocardial infarction, congestive heart failure, peripheral vascular disease, cerebrovascular disease, mild liver disease, diabetes, moderate or severe renal disease, and diabetes without chronic complication) cancer-related treatment (surgery, radiation therapy, chemotherapy, and target therapy), anti-hypertensive medication (calcium channel blockers, beta blockers, diuretics, and other-classes antihypertension) and co-medication within 6 months before and after cancer diagnosis (metformin, non-steroidal anti-inflammatory drugs, statins, bisphosphonates, and antithrombotic agents).

^b^SIPTW=Stabilize inverse probability of treatment weighting

^c^The post-diagnosis period was defined as the period within 6 months after cancer diagnosis.

^d^Cumulative defined daily dose (cDDD) was calculated as each patient received DDD of ACEIs/ARBs at the post-diagnosis period (within 6 months after cancer diagnosis).

^e^Low-dose group defined as patients received the cDDD of ACEIs/ARBs at the post-diagnosis period was lower than the median cDDD of ACEIs/ARBs. The median cDDD of ACEIs/ARBs at the post-diagnosis period in esophageal and gastric cancer patients were 113.5 and 122, respectively.

^f^High-dose group defined as patients received the cDDD of ACEIs/ARBs at the post-diagnosis period was at least the median cDDD of ACEIs/ARBs.

Table S7: Subgroup and sensitivity analysis of all-cause mortality in esophageal/gastric cancer

| Esophageal cancer | Adjusted HR^a^  with SIPTW^b^ (95% CI) | p-value | Forest plot |
| --- | --- | --- | --- |
| **Stage of cancer** |  |  | 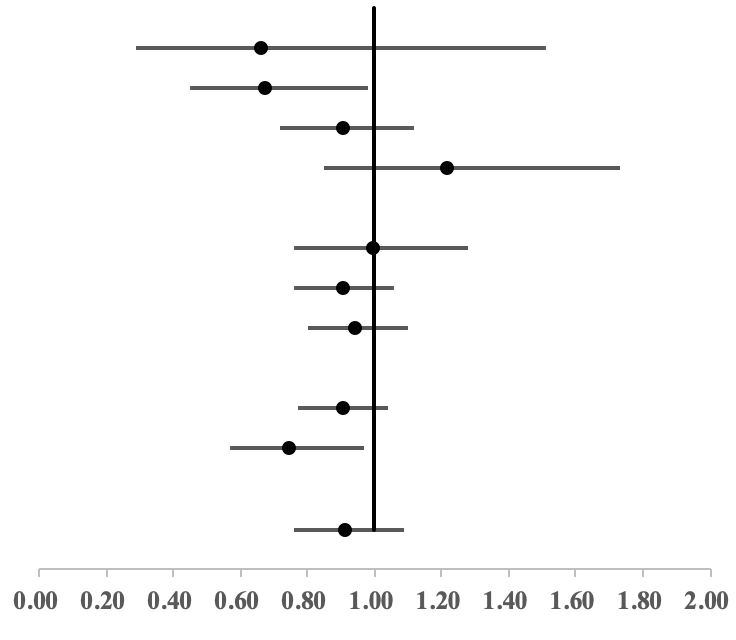  **ACEIs/ARBs non-user**  **better**  **ACEIs/ARBs user**  **better** |
| Stage ≤1 | 0.66 (0.29-1.51) | 0.329 |  |
| Stage =2 | 0.67 (0.45-0.98) | 0.038 |  |
| Stage =3 | 0.90 (0.72-1.12) | 0.341 |  |
| Stage =4 | 1.21 (0.85-1.73) | 0.299 |  |
| **Cancer treatment** |  |  |  |
| Surgery | 0.99 (0.76-1.28) | 0.928 |  |
| Radiation therapy | 0.90 (0.76-1.06) | 0.194 |  |
| Chemotherapy | 0.94 (0.80-1.10) | 0.424 |  |
| **Others** |  |  |  |
| Squamous cell carcinoma^c^ | 0.90 (0.77-1.04) | 0.138 |  |
| Specific comorbidities^d^ | 0.74 (0.57-0.97) | 0.029 |  |
| **Sensitivity** **analysis** |  |  |  |
| Follow-period > 6months^e^ | 0.91 (0.76-1.09) | 0.318 |  |
|  |  |  |  |
|  |  |  |  |
| Gastric cancer |  |  |  |
| **Stage of cancer** |  |  | 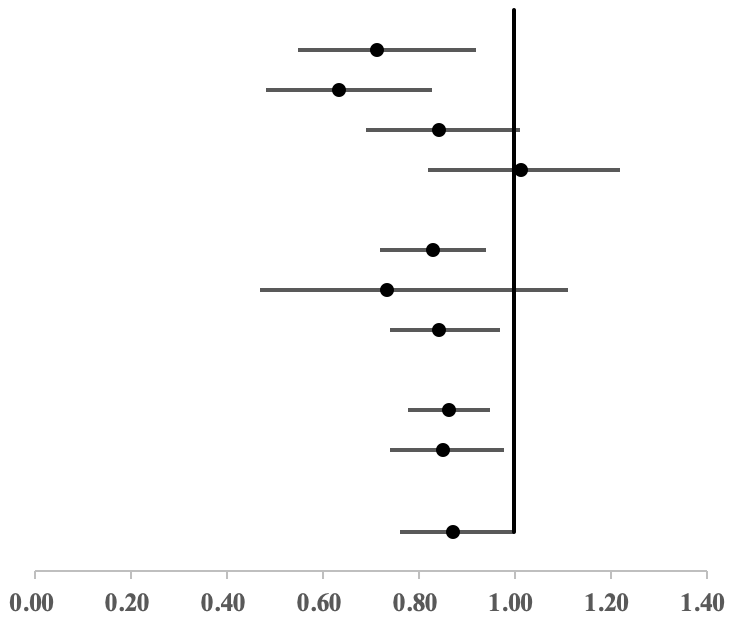  **ACEIs/ARBs user**  **better**  **ACEIs/ARBs non-user**  **better** |
| Stage ≤1 | 0.71 (0.55-0.92) | 0.010 |  |
| Stage =2 | 0.63 (0.48-0.83) | 0.001 |  |
| Stage =3 | 0.84 (0.69-1.01) | 0.067 |  |
| Stage =4 | 1.01 (0.82-1.22) | 0.963 |  |
| **Cancer treatment** |  |  |  |
| Surgery | 0.83 (0.72-0.94) | 0.005 |  |
| Radiation therapy | 0.73 (0.47-1.11) | 0.139 |  |
| Chemotherapy | 0.84 (0.74-0.97) | 0.014 |  |
| **Others** |  |  |  |
| Adenocarcinoma^f^ | 0.86 (0.78-0.95) | 0.003 |  |
| Specific comorbidities^d^ | 0.85 (0.74-0.98) | 0.027 |  |
| **Sensitivity** **analysis** |  |  |  |
| Follow-period > 6months^e^ | 0.87 (0.76-1.00) | 0.049 |  |
|  |  |  |  |
|  |  |  |  |

^a^Adjusted variables included age, gender, year of diagnosis, histology, cancer stage, geographic region, comorbidities (myocardial infarction, congestive heart failure, peripheral vascular disease, cerebrovascular disease, mild liver disease, diabetes, moderate or severe renal disease, diabetes without chronic complication) cancer-related treatment (surgery, radiation therapy, chemotherapy, target therapy), anti-hypertensive medication (calcium channel blockers, beta blockers, diuretics, other-classes antihypertension) and co-medication within 6 months before and after cancer diagnosis (metformin, non-steroidal anti-inflammatory drugs, statins, bisphosphonates, antithrombotic agents).

^b^SIPTW=Stabilize inverse probability of treatment weighting

^c^Restricted to patients with esophageal squamous cell carcinoma.

^d^Restricted to patients with myocardial infarction, congestive heart disease, diabetes mellitus or diabetes mellitus with complication in the year prior to the cancer diagnosis.

^e^Restricted to patients who live longer than 6 months after cancer diagnosis.

^f^Restricted to patients with gastric adenocarcinoma.


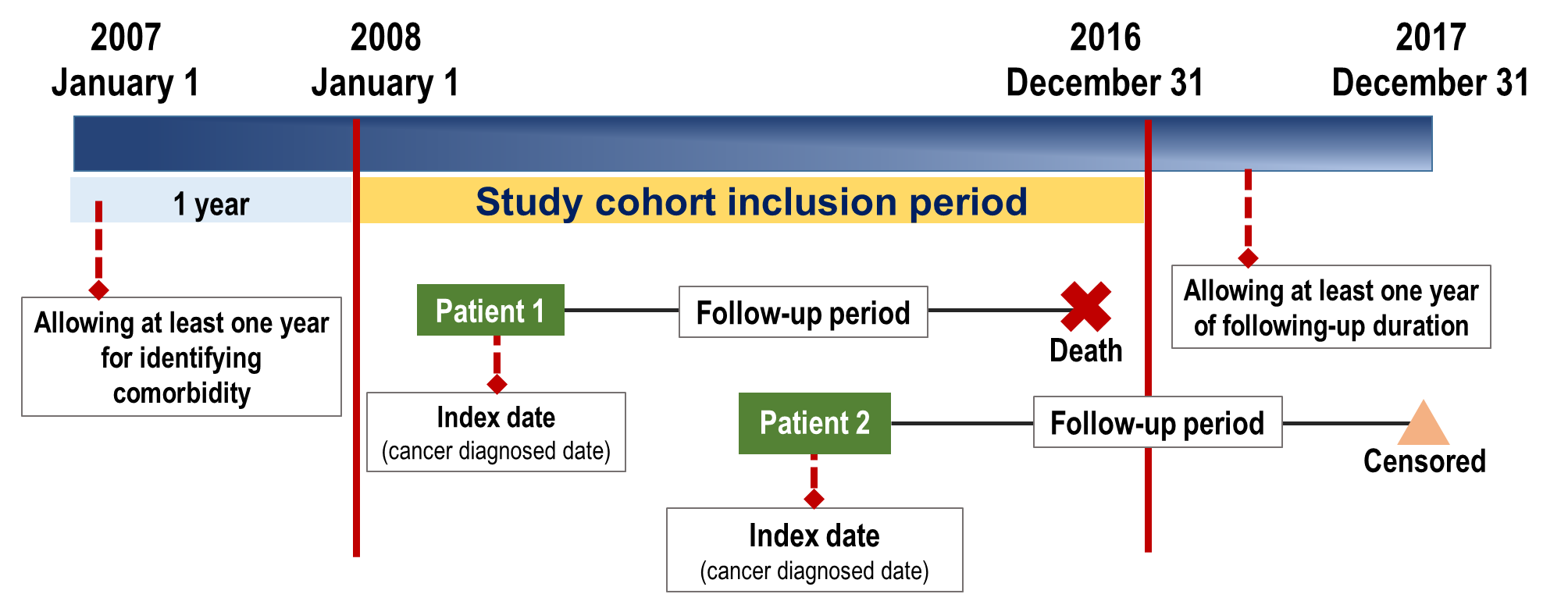


Figure S1: Diagram of study time frame and demonstration of patients’ follow-up
